# Virginia Medical Journal

**Published:** 1858-07

**Authors:** 


### Virginia Medical Journal.

To our friends who are looking out for an addition to their stock of periodical Medical Literature, we would desire to commend in the highest terms, the "Virginia Medical Journal," as being one to which they may always look with confidence, for something new and substantial.

By this allusion, we of course mean no invidious comparison, but not being connected with any Medical School, and relying alone for support upon its merits, we should like to see it, as it deserves, in general circulation throughout the Southern country, to whose medical literature, it is certainly an *honor.* It is published in Richmond, Va., and edited by Drs. McCaw & Otis.

